# Further Delineation of the Spectrum of XMEN Disease in Six Chinese Pediatric Patients

**DOI:** 10.3389/fgene.2022.768000

**Published:** 2022-01-25

**Authors:** Xiaomin Peng, Yi Lu, Huijun Wang, Bingbing Wu, Mingyu Gan, Suzhen Xu, Deyi Zhuang, Jianshe Wang, Jinqiao Sun, Xiaochuan Wang, Wenhao Zhou

**Affiliations:** ^1^ Center for Molecular Medicine of Children’s Hospital of Fudan University and National Children’s Medical Center, Institutes of Biomedical Sciences, Fudan University, Shanghai, China; ^2^ Center for Pediatric Liver Diseases, Children’s Hospital of Fudan University and National Children’s Medical Center, Shanghai, China; ^3^ Center for Molecular Medicine, Key Laboratory of Birth Defects, Pediatrics Research Institute, Children’s Hospital of Fudan University and National Children’s Medical Center, Shanghai, China; ^4^ Department of Pediatrics, Xiamen Children’s Hospital, Xiamen, China; ^5^ Department of Clinical Immunology, Children’s Hospital of Fudan University and National Children’s Medical Center, Shanghai, China

**Keywords:** *MAGT1* gene, XMEN, elevated liver enzymes, immunodeficiency, genetic testing

## Abstract

X-linked MAGT1 deficiency with increased susceptibility to EBV-infection and N-linked glycosylation defect (XMEN) disease is a primary immunodeficiency caused by loss-of-function variants in the *MAGT1* gene. Only two patients from one family have been diagnosed with XMEN in China. In this study, we retrospectively analyzed the genetic, clinical, and immunological characteristics of six pediatric patients in a Chinese cohort. Medical records were retrieved, immunological phenotypes were assessed, and infectious microbes in patients were detected. Six male patients (mean age, 6.3 years) from five unrelated families were genetically diagnosed as XMEN. Five patients presented with a major complaint of elevated liver enzymes, while one patient was referred for recurrent fever, cough and skin rash. Five patients developed EBV viremia, and one patient developed non-Hodgkin’s lymphoma. Histopathological findings from liver biopsy tissues showed variable hepatic steatosis, fibrosis, inflammatory infiltration, and glycogenosis. Immune phenotypes included CD4 T-cell lymphopenia, elevated B cells, inverted CD4/CD8 ratios, and elevated αβDNTs. No pathogenic microbes other than EBV were identified in these patients. This study reports the clinical and molecular features of Chinese patients with XMEN. For patients with transaminase elevation, chronic EBV infection and EBV-associated lymphoproliferative disease, the possibility of XMEN should be considered in addition to isolated liver diseases.

## Introduction

The magnesium transporter 1 (*MAGT1*) gene (OMIM: 300715) is located on chromosome Xq21.1 and comprises 10 exons. Its largest transcript variant, NM_032121.5, encodes a 367 amino acid protein conserved in eukaryotes (Zhou and Clapham 2009). MAGT1 protein consists of a signal peptide, a thioredoxin (TRX) domain that contains a bi-cysteine motif (CXXC), and four transmembrane (TM) regions (Zhou and Clapham 2009; Matsuda-Lennikov et al., 2019). As an Mg^2+^-specific ion transporter, MAGT1 regulates free intracellular Mg^2+^ and mediates a transient influx of Mg^2+^ during T-cell activation. MAGT1 shares a 66% amino acid identity with TUSC3 and is a non-catalytic subunit of the oligosaccharyltransferase (OST) complex, facilitating glycosylation in a STT3B-dependent manner (Zhou and Clapham 2009; Cherepanova et al., 2014). MAGT1 also promotes STT3B-dependent N-linked glycosylation (NLG) of a subset of glycoproteins crucial for proper immune function (Cherepanova et al., 2014; Matsuda-Lennikov et al., 2019; Ravell et al., 2020b).

Hemizygous loss-of-function variants in the *MAGT1* gene lead to a rare primary immunodeficiency known as X-linked MAGT1 deficiency with increased susceptibility to Epstein-Barr virus (EBV) infection and N-linked glycosylation defect (XMEN, previously known as X-linked immunodeficiency with magnesium defect, EBV infection, and neoplasia) disease (Ravell et al., 2020a). MAGT1 deficiency was initially recognized as a novel combined immunodeficiency because the affected patients showed defective development and function of T cells associated with chronic active EBV infections (Li et al., 2011). It has been demonstrated that loss of MAGT1 decreases Mg^2+^ influx as well as the expression of NKG2D in natural killer (NK) cells and T lymphocytes, which are vital for antiviral and antitumor cytotoxicity (Chaigne-Delalande et al., 2013; Li et al., 2014). *MAGT1*-knockout mice also showed impaired Mg^2+^ homeostasis and increased numbers and hyper-activation of B cells, although there were no defects in T-cell activation (Gotru et al., 2018). Further observations expanded the phenotypic spectrum of MAGT1 deficiency to intellectual and developmental disability caused by abnormal glycosylation (Blommaert et al., 2019; Ravell et al., 2020b). Clinical manifestations and severity of XMEN are highly variable, including recurrent ear and sinus infections, chronic EBV infections, lymphadenopathy, molluscum contagiosum, autoimmune cytopenia, EBV-positive lymphoma, and central nervous system abnormalities (Ravell et al., 2020b). Elevated liver enzymes and autoimmune hepatitis were first reported in an adolescent boy who presented with severe autoimmune disorders mimicking autoimmune lymphoproliferative disease (ALPS) (Patiroglu et al., 2015). Recently, Ravell *et al.* found noninfectious liver abnormalities with evidence of defective glycosylation instead of autoimmune hepatitis in patients with XMEN and further distinguished XMEN and ALPS using leukocyte surface marker clusters (Ravell et al., 2020b). Therefore, XMEN has been demonstrated to be a selective congenital disorder of glycosylation that predominantly manifests as immunodeficiency (Ravell et al., 2020a).

Forty-five unique male patients have been reported since XMEN was first reported in 2011 (Li et al., 2011; Chaigne-Delalande et al., 2013; Li et al., 2014; Dhalla et al., 2015; Patiroglu et al., 2015; Brigida et al., 2017; He et al., 2018; Blommaert et al., 2019; Dimitrova et al., 2019; Ravell et al., 2020b; Hoyos-Bachiloglu et al., 2020; Klinken et al., 2020; Brault et al., 2021). However, only two cases from one family have been reported in China to date. These two cases suffered from recurrent upper respiratory tract infections and sinusitis (He et al., 2018). In this study, we reported six patients in five unrelated Chinese families with pathogenic/likely pathogenic (P/LP) variants in *MAGT1*, which lead to a diagnosis of XMEN. Interestingly, most patients presented with symptoms of elevated transaminase levels and chronic EBV infections.

## Materials and Methods

### Patients

We retrospectively analyzed six pediatric patients who were diagnosed with XMEN in the genetic laboratory of Children’s Hospital of Fudan University from January 2019 to January 2021. Electronic medical records were retrieved and reviewed. Archived diagnostic slides of liver biopsies were re-assessed by an experienced clinical pathologist. This study was approved by the Ethics Committee of Children’s Hospital of Fudan University (2015–130). Written informed consent was obtained from the patients’ guardians.

### Genetic Testing and Data Analysis

Peripheral blood samples were obtained from the probands and their parents. Genomic DNA was isolated from peripheral blood mononuclear cells using Qiagen genomic DNA extraction kit (Qiagen, Hilden, Germany). Extracted genomic DNA was enriched using the Agilent ClearSeq Inherited disease Kit (Agilent Technologies, Santa Clara, CA, United States) including 2,742 genes for clinical exome sequencing (CES), or the Agilent SureSelect All Exon Human V5 Kit for exome sequencing (ES) as previously described (Dong et al., 2020; Yang et al., 2020). After amplification using polymerase chain reaction (PCR), DNA libraries were sequenced on an Illumina HiSeq2500 platform (Illumina, San Diego, CA, United States) to obtain 150 bp paired-end sequencing reads.

Sequences were aligned with the human genome assembly GRCh37/hg19 using Burrows-Wheeler Aligner (BWA) version 0.7.15-r1140 (Li and Durbin 2009). Subsequent processing of sorting, merging and removing duplicated BAM files was performed using SAMtools (v.1.8) (Li et al., 2009) and Picard tools (v.2.20.1). Variant callings, including single-nucleotide variations (SNV) and small indels, were obtained using the GATK tool and best practice pipelines with default parameters. Home-modified CANOES was applied to detect CNVs from exon-based read counts (Dong et al., 2020). Candidate pathogenic variants in *MAGT1* were validated using Sanger sequencing. The nonsense-mediated mRNA decay (NMD) efficacy of novel *MAGT1* variants was predicted using an online NMDective resource (Lindeboom et al., 2019). Pathogenicity was defined based on the standards and guidelines of the American College of Medical Genetics and Genomics (ACMG) (Richards et al., 2015) and the ClinGen Sequence Variant Interpretation (SVI) Working Group.

### Identification of Pathogenic Microbes

For metagenomic sequencing, DNA was extracted from liver biopsy tissues of patient 4 and 5 (P4 and P5, respectively) using a QIAamp UCP Pathogen Mini Kit (Qiagen, Hilden, Germany). The sequencing library was constructed using KAPA Hyper Prep Kit (Kapa Biosystems, Wilmington, MA, United States). Libraries were then pooled and sequenced on the Illumina HiSeq 2000 platform (Illumina, San Diego, CA, United States) using a 75-cycle single-end index sequencing kit. At least 20 million single-end reads were obtained from each sample. Reads were quality trimmed with a 10-base sliding window. Trimming was performed when the average base quality dropped below 15 using Trimmomatic v0.39 (Bolger et al., 2014). Reads shorter than 40 bases and human reads were subsequently removed. The remaining reads were aligned to the microorganism reference genome database using Centrifuge v1.0.3. Reads with an alignment rate >70% were retained. In addition, reads that aligned to multiple different species genomes were excluded.

To detect infectious pathogens including EBV in the blood, DNA was extracted from whole blood of these six patients using QIAamp UCP Pathogen Mini Kit. The diluted pre-amplification products were mixed with TaqMan OpenArray Real-Time PCR Master Mix and automatically dispensed on the OpenArray plate. Real-time PCR of the OpenArray plate was performed using the QuantStudio 12K Flex instrument (Thermo Fisher Scientific, Waltham, MA, United States) following the manufacturer’s instructions. Non-template control (NTC) was added to each batch of real-time PCR. Cq confidence, amplification score, and Ct value was calculated using the Thermo Fisher Scientific’s proprietary algorithm. Amplifications with an amplification score >1.2 and Cq confidence >0.7 were considered of sufficient quality and retained for the following analyses. Positive detection was identified only when all the replicated assays were qualified amplified and no amplification was detected in the NTC.

### Immunological Assays

Routine blood counts and immunological phenotypes were evaluated as previously reported (Wang et al., 2018). The levels of serum immunoglobulins, including IgG, IgA, IgM, and IgE, were detected using nephelometry, while lymphocyte subsets were analyzed using a FACSCalibur flow cytometer (Becton Dickinson, Franklin Lakes, NJ, United States). The following validated antibodies were used for flow cytometry: anti-CD3 (UCHT1), anti-CD4 (RPA-T4), anti-CD8 (RPA-T8), anti-CD27 (M-T271), anti-TCRαβ (T10B9.1A-31), anti-TCRγδ (B1), anti-CD45RA (HI100), anti-CD19 (HIB19), anti-CD24 (ML5), anti-CD38 (HIT2), and anti-IgD (IA6–2) (all from BD Biosciences, San Jose, CA, United States).

## Results

### Clinical Manifestations

Six male patients from five unrelated families were clinically diagnosed with XMEN based on genetic testing results, laboratory studies, and clinical manifestations. The mean age at diagnosis was 6.3 years (range, 19 months to 10-years-7-months). All patients were born to healthy, non-consanguineous parents. P4 and P5 were from the same family, and the maternal uncle died of lymphoma at 10 years of age.

**FIGURE 1:**
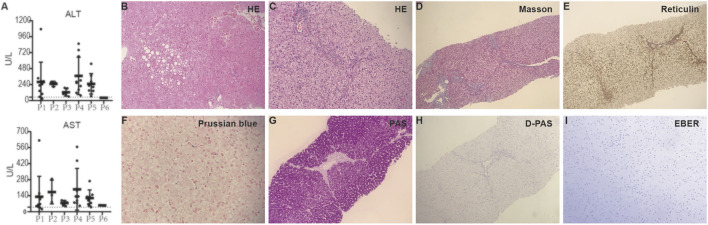
Abnormal liver function tests and pathological findings of liver tissues in patients with XMEN disease in this study. **(A)** ALT (upper panel) and AST (lower panel) levels in all patients with a normal range (the dotted line, except for P6) and patients’ identities (shown on the *X*-axis). **(B–H)** Representative images depicting histopathologic findings from liver biopsy tissues of patients. **(B & C)** Hematoxylin and eosin staining results showing mild hepatocyte swelling, hepatosteatosis, periportal fibrosis, inflammatory infiltrations and sinusoidal iron deposits, ×200. **(D)** Masson’s Trichrome staining results showing periportal fibrosis and bridging fibrosis, ×100. **(E)** Reticulin staining results showing preserved despite focal disrupted reticulin framework, ×100. **(F)** Prussian blue staining results showing mild iron deposition in hepatocytes, ×100. PAS **(G)** and D-PAS **(H)** staining depicting diffuse glycogenosis, ×100. **(I)** Liver tissues from patient 1 were negative for EBER staining, ×200. *P*, patient.

Interestingly, five of six patients (except for P6) were referred to the center for liver diseases in our hospital because of abnormal liver function tests (LFTs) (Figure 1A). These five patients were negative for conventional autoantibodies associated with autoimmune hepatitis, including anti-ANA, anti-LKM, anti-AMA, and anti-SMA. Liver diseases such as intrahepatic cholestasis, biliary atresia and Wilson’s disease were excluded because the five patients had normal levels of serum γ-glutamyltransferase (GGT), muscle creatine phosphokinase (CPK), bilirubin, bile acid, and ceruloplasmin. However, P6 presented with recurrent fever, cough, oral ulcers, and urticaria; these symptoms lead to clinical suspicion of an immunodeficiency disease. He developed neutropenia (1.51 × 10^9^ cells/L at the last evaluation, range = 1.18–1.74 × 10^9^ cells/L). Two patients (P4 and P5) underwent brain MRI, and showed no specific neurological findings, although half of the patients had cavum septum pellucidum in a recent study (Ravell et al., 2020b). Intellectual disability or facial dysmorphism was not observed in any of these patients.

Recurrent upper respiratory tract infections were observed in P1, P2, P3 and P4. Aphthous ulcers and sinusitis were observed in P4 and P5, rhinitis in P4, and otitis media in P5. P2 experienced an episode of erythema multiforme during pneumonia. Neck and submandibular lymphadenopathy was observed in P1 and P2, respectively. Generalized lymphadenopathy and lymphadenitis occurred in both P4 and P5. Moreover, P1 developed a painless mass on the left lower lip at 4-years-6-months and was diagnosed with mature B-cell non-Hodgkin’s lymphoma. He also had a history of suppurative osteomyelitis of the right knee and intrahepatic nodules and presented with mild hepatosplenomegaly. Abdominal ultrasonography revealed increased spleen thickness and splenomegaly in P4 and P5.

Four patients (P1, P3, P4, and P5) underwent percutaneous core needle liver biopsy. Histopathological analysis revealed varying degrees of liver damage (Figures 1B–H). P1 had mild steatosis, mild sinusoidal iron deposits, and lymphocyte infiltration. P3 showed moderate hepatic steatosis, small vacuoles and eosinophilic bodies in the hepatocyte cytoplasm, and glycogenated hepatocyte nuclei. P4 and P5 showed a similar pattern of injury, including hepatocyte swelling, periportal fibrous proliferation, bridging fibrosis, inflammatory cell infiltration, and glycogenosis. The liver specimen from P1 was EBV-encoded small RNA (EBER) negative (Figure 1I). According to metagenomic sequencing, the liver specimens from P4 and P5 were negative for EBV sequences. All patients, except P6, had EBV infection, confirmed through the detection of EBV-specific antibodies or EBV DNA in blood samples. Probe-based qPCR of whole blood samples revealed that no patient had cytomegalovirus (CMV), adenovirus, or herpes simplex virus type 1 (HSV-1). Infection studies showed negative results for hepatitis A, hepatitis B, hepatitis C, CMV, rubella virus, parvovirus B19, and human immunodeficiency virus (HIV) in all patients, though P1 had a history of transient HSV-1 infection. The clinical and genetic characteristics of these six patients are summarized in Table 1 and Supplementary Material.

**TABLE 1:** Clinical and genetic characteristics of six XMEN patients in this study.

	Patient 1	Patient 2	Patient 3	Patient 4	Patient 5	Patient 6
Sex	male	male	male	male	male	male
Age at onset	4 y6 m	3 y2 m	4 y3 m	5 y	7 y2 m	13 m
Age at genetic testing	10 y7 m	4 y8 m	6 y3 m	6 y2 m	8 y5 m	19 m
Major complaint	abnormal LFTs for 5 years and intrahepatic nodules for 1 y	abnormal LFTs for more than 1 y	abnormal LFTs for 6 m	abnormal LFTs for 1 y	abnormal LFTs for 1 y	recurrent upper respiratory tract infection
*MAGT1* variants						
chromosome location (hg19)	chrX:77111082	chrX:77111053	chrX:77150909	chrX:77096749	chrX:77096749	chrX:77112895
cDNA (NM_032121.5)	exon6:c.774dupT	exon6:c.803G > A	exon1:c.95dupA	exon8:c.991C > T	exon8:c.991C > T	exon4:c.586G > T
protein	p.(Val259CysfsTer21)	p.(Trp268Ter)	p.(Asn32LysfsTer40)	p.(Arg331Ter)	p.(Arg331Ter)	p.(Glu196Ter)
inheritance	*De novo*	*De novo*	Maternal	Maternal	Maternal	Maternal
classification	pathogenic (PVS1+PM2_Supporting + PM6)	pathogenic (PVS1+PM2_Supporting + PM6)	likely pathogenic (PVS1+PM2_Supporting)	likely pathogenic (PVS1+PM2_Supporting)	likely pathogenic (PVS1+PM2_Supporting)	likely pathogenic (PVS1+PM2_Supporting)
ClinVar accession number	SCV001827201.1	SCV001827202.1	SCV001827203.1	—	—	SCV001827204.1
Liver function tests						
ALT (U/L)	30–1072 (9–50)	205–280 (9–50)	59–189.8 (9–50)	74.5–853.1 (9–50)	65–551 (9–50)	38–42 (0–40)
AST (U/L)	23–623 (15–40)	85–287.4 (15–40)	48–99.6 (15–40)	52.20–566.8 (15–40)	40–268 (15–40)	53–58 (0–40)
GGT (U/L)	21.3–37.9 (8–57)	16–39.2 (8–57)	25–41.4 (8–57)	34–202 (8–57)	23.6–44.6 (8–57)	12–15 (7–50)
CPK (U/L)	80–117 (0–164)	78–97 (0–164)	147–176 (0–164)	125–221 (0–164)	134–148 (0–164)	NA
Liver histopathology	mild steatosis, sinusoidal iron deposits	NA	moderate steatosis, glycogenosis	bridging fibrosis, glycogenosis	bridging fibrosis, glycogenosis	NA
Neutropenia	+	-	-	-	-	+
Sinopulmonary infections	+	+	+	+	+	+
Lymphadenopathy	+	+	-	+	+	-
Hepatosplenomegaly	+	-	-	+	+	-
EBV-DNA in PBMC (copies/ml) ^†^	8.02 × 10^4^	8.12 × 10^5^	5.83 × 10^3^	1.19×10^5^	1.52×10^5^	-
Pan-pathogen detection	EBV	EBV	EBV	EBV	EBV	negative
Lymphoma (age)	non-Hodgkin’s lymphoma (5y2m)	no	no	no	no	no
NKG2D expression	NA	NA	NA	NA	NA	NA
Immunologic data						
IgG (g/L)	7.50 (6.09–12.85)	6.13 (6.94–16.2)	10.50 (4.95–12.74)	5.60 (4.95–12.74)	5.40 (6.09–12.85)	7.6 (6.94–16.2)
IgA (g/L)	0.64 (0.52–2.16)	0.12 (0.68–3.78)	1.19 (0.33–1.89)	0.35 (0.33–1.89)	0.21 (0.52–2.16)	0.165 (0.82–4.53)
IgM (g/L)	2.55 (0.67–2.01)	0.83 (0.6–2.63)	1.10 (0.65–2.01)	1.24 (0.65–2.01)	0.91 (0.67–2.01)	1.12 (1–3)
total T cells (%)	61.20 (64–73)	72.68 (59–84)	68.51 (64–73)	44.72 (64–73)	59.28 (64–73)	47.12↑*
total T cells (cells/μL)	1755.3 (1325–2276)	NA	2042.3 (1424–2664)	1155.3 (1424–2664)	1866.2 (1325–2276)	4208 (1563–3929)
CD4 T cells (%)	20.15 (29–36)	26.17 (31–60)	34.20 (29–36)	18.23 (29–36)	27.84 (29–36)	11.20*
CD4 T cells (cells/μL)	578.09 (531–1110)	NA	1019.65 (686–1358)	470.99 (686–1358)	876.31 (531–1110)	916 (738–2001)
CD8 T cells (%)	31.65 (24–34)	40.20 (13–38)	25.59 (24–34)	14.82 (24–34)	21.66 (24–34)	25.16↑*
CD8 T cells (cells/μL)	907.87 (480–1112)	NA	762.93 (518–1125)	382.90 (518–1125)	681.76 (480–1112)	2058 (532–1549)
CD4/CD8	0.59 (0.81–1.66)	0.65 (0.9–3.6)	1.34 (0.87–1.94)	1.23 (0.87–1.94)	1.29 (0.81–1.66)	0.45 (0.90–2.13)
total B cells (%)	24.95 (14–21)	17.22 (7–22)	14.27 (14–21)	42.89 (14–21)	28.94 (14–21)	37.08↑*
total B cells (cells/μL)	715.54 (216–536)	NA	425.46 (280–623)	1107.97 (280–623)	911.03 (216–536)	3311 (261–960)
NK cells (%)	13.37 (11–23)	4.18 (6–27)	16.07 (11–23)	11.29 (11–23)	10.15 (11–23)	11.21↑*
NK cells (cells/μL)	383.54 (246–792)	NA	479.09 (258–727)	291.76 (258–727)	319.66 (246–792)	1001 (197–786)
αβDNT (%)	3.95 (0.61–2.31)	NA	NA	NA	NA	NA
Follow-up						
age at last follow-up	13 y	6 y6 m	7 y5 m	7 y	9y3m	26 m
outcome	remission of lymphoma, AS	AS	AS	AS	AS	AS

The number in the round bracket presents the age-specific reference values according to reference values for liver enzyme levels or peripheral blood lymphocyte subsets of healthy children in China; ^†^ prior to treatment; *the reference values are not available; NA, data not available; AS, alive symptomatic.

### Genetic Testing Results

**FIGURE 2:**
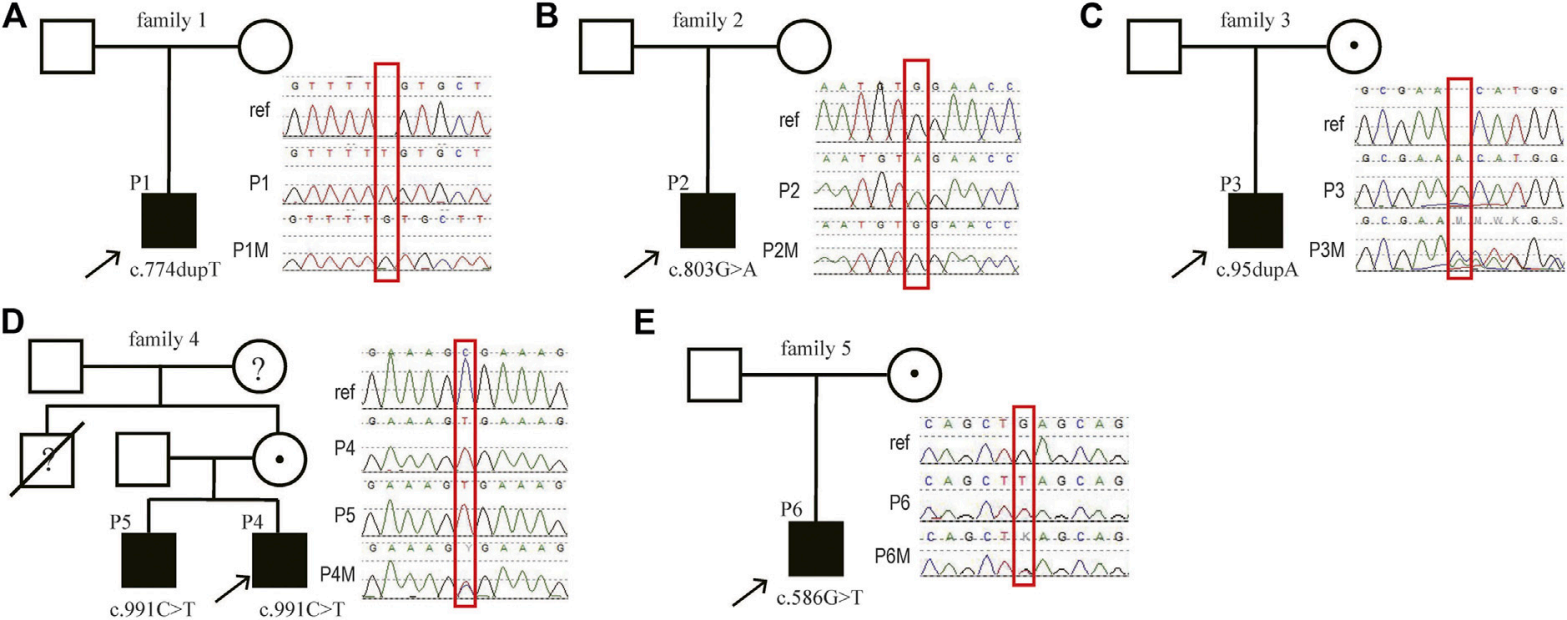
Identification and validation of the *MAGT1* variants in six patients with XMEN disease in this study. Pedigrees of patient 1 **(A)**, patient 2 **(B)**, patient 3 **(C)**, patient 4 and 5 **(D)** and patient 6 **(E)** carrying disease-causing variants in the *MAGT1* gene. *P*, patient; *M*, mother; *ref*, reference sequence.

**FIGURE 3:**
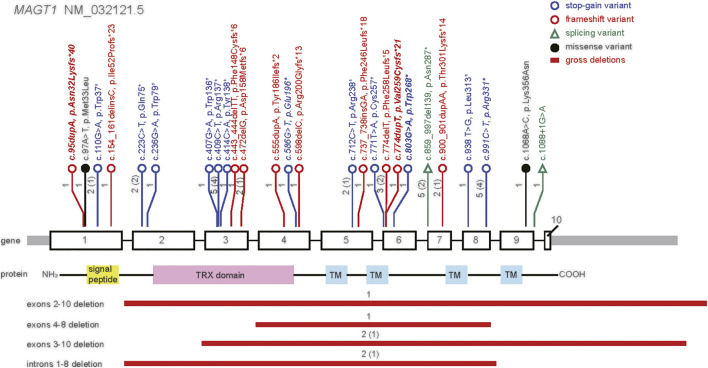
Distribution of disease-causing variants in *MAGT1* gene in previous literatures and the present study. MAGT1 protein consists of a signal peptide, a thioredoxin (TRX) domain and four transmembrane (TM) regions. Variants identified in this study are in italics, and novel variants are in bold. The number of patients carrying each variant is labeled with family numbers in round brackets.

Three of the six patients had novel variants in *MAGT1* (NM_032121.5, P1: c.774dupT; P2: c.803G > A and P3: c.95dupA) (Table 1; Figures 2, 3). The variants found in P1 and P2 were not detected in their mothers, indicating that they were *de novo* variants. P1 underwent proband CES testing, and his mother underwent Sanger sequencing. Genetic studies revealed a hemizygous variant c.774dupT in *MAGT1* in P1; this variant is predicted to cause a frameshift with premature termination of the protein (p. (Val259CysfsTer21)). Furthermore, this variant was not detected in his mother through Sanger sequencing. P2 was found to harbor a *de novo* nonsense variant in *MAGT1* (c.803G > A); this variant is predicted to result in a stop codon with premature termination of the protein (p. (Trp268Ter)). Trio-ES was conducted in family 3 and revealed a frameshift variant (c.95dupA, p. (Asn32LysfsTer40)) in *MAGT1* in the proband inherited from his mother. Trio-ES showed a hemizygous nonsense variant (c.586G > T, p. (Glu196Ter)) in *MAGT1* in P6 inherited from his mother. P4 and P5 were found to inherit a previously reported disease-causing variant of c.991C > T (Blommaert et al., 2019; Ravell et al., 2020b; Brault et al., 2021) from their mother, introducing an amino acid exchange from arginine to a stop codon at position 331 (p. (Arg331Ter)). The maternal uncle died at 10 years of age without diagnosis (Figure 2).

None of these novel variants in *MAGT1* has been previously reported in known public databases (gnomAD and 1000 Genomes) or our in-house database (more than 40,000 individuals). The loss-of-function intolerance (pLI) score for *MAGT1* is 0.96, and the observed/expected (o/e) ratio is 0.07; suggesting that *MAGT1* is highly intolerant to loss-of-function variants. Most reported disease-causing mutations in *MAGT1* are loss-of-function variants (Li et al., 2011; Chaigne-Delalande et al., 2013; Li et al., 2014; Dhalla et al., 2015; Patiroglu et al., 2015; Brigida et al., 2017; He et al., 2018; Blommaert et al., 2019; Dimitrova et al., 2019; Ravell et al., 2020b; Hoyos-Bachiloglu et al., 2020; Klinken et al., 2020; Brault et al., 2021). All these novel variants occur upstream to the last *MAGT1* exon. In addition, all variants except c.95dupA are predicted to efficiently trigger NMD according to the results of the NMDective algorithm. However, the adjacent nonsense variant c.110G > A has been reported in patients diagnosed with XMEN who presented with EBV viremia, inverted CD4/CD8 ratios, and decreased NKG2D expression (Li et al., 2014; Ravell et al., 2020b). According to the ACMG guidelines and the ClinGen SVI recommendations, these novel variants are classified as P/LP variants.

### Autoimmune Characteristics and Immune Phenotype Evaluation

Routine blood analysis revealed transient mild neutropenia in P1 and P6, and lymphocytosis in P2 and P6. No other blood system abnormalities were observed in these patients (data not shown). IgA level was decreased in P2, P5, and P6, whereas IgG level was slightly decreased in P2 and P5. CD4 T-cell lymphopenia was observd in four patients (P1, P2, P4, and P5), although opportunistic infections were rarely observed. Three patients (P1, P2, and P6) had inverted CD4/CD8 T-cell ratios, and four patients (P1, P4, P5, and P6) had increased numbers of B cells. NK-cell counts were slightly decreased in P2 and P5. P1 underwent T-cell and B-lymphocyte subpopulation analysis, which revealed increased numbers of CD4^–^CD8^–^TCRαβ^+^ T cells (αβDNTs) and naïve cytotoxic T cells (CD8^+^ CD27^+^CD45RA^+^) (Table 1 and Supplementary Table S1). Unfortunately, NKG2D expression on NK and CD8 cells was not evaluated. No other significant abnormalities were found in the routine assessment of the immune phenotypes.

### Treatment and Outcome

P1 received five courses of chemotherapy according to a modified B-NHL-BFM 95 protocol for non-Hodgkin’s lymphoma. Rituximab was added in his last chemotherapy session considering bone marrow involvement, and the patient achieved complete remission of lymphoma for 7 years. All six patients underwent prophylactic antibiotic and symptomatic treatment. None of them received intravenous immunoglobulin (IVIG) or hematopoietic stem cell transplantation (HSCT). All patients were in stable condition during follow-ups. Specifically, all patients had sinopulmonary infections for 5–10 times per year; all patients except P6 had persistent EBV viremia accompanied by fluctuated liver transaminase levels.

## Discussion

In this study, we reported five MAGT1 deficient patients with a major complaint of abnormal LFTs. P1 had persistently elevated liver enzymes prior to and throughout chemotherapy for non-Hodgkin’s lymphoma, and the abnormal LFTs had been detected for the past 5 years. P2, P3, P4 and P5 presented with mild to moderate elevated serum transaminases (ALT and AST) levels and mostly normal CPK and GGT levels. The symptom of elevated LFTs was first described in a 15-year-old Turkish boy in 2015, who presented with frequent sinopulmonary infections and severe autoimmune disorders, and was finally diagnosed with XMEN (Patiroglu et al., 2015). However, autoimmune hepatitis was excluded from our patients because detection results of conventional autoantibodies were negative. Infection studies showed negative results for hepatitis A, hepatitis B, and hepatitis C. EBER staining of the liver tissue from P1 was negative. The EBV sequence could not be detected in the liver tissues from P4 and P5 using metagenomic sequencing. Liver biopsies from four patients with EBV infection showed no evidence of EBV-associated hepatitis. Instead, histopathology revealed a non-specific pattern of injury with variable degrees of hepatosteatosis, inflammatory infiltrates, fibrosis, and glycogenosis; this finding is consistent with that of a previous study (Ravell et al., 2020b). In the largest XMEN cohort study, transient and asymptomatic elevations in ALT and AST levels were observed in all 23 patients, whereas elevation in serum CPK was detected in 12 cases (Ravell et al., 2020b). Both EBV-infected and EBV-negative patients exhibited similar histopathological patterns and liver enzyme levels (Ravell et al., 2020b). Blommaert *et al.* (Blommaert et al., 2019) also demonstrated that MAGT1-deficient patients have a defect in glycosylation with enhanced expression of the MAGT1 homolog protein TUSC3. Taken together, these findings suggest that liver diseases in XMEN are related to selective NLG defects but not to EBV infections.

All patients in our study, except P6, presented with EBV infection. Chronic EBV infections were common in XMEN patients because of the defective expression of NKG2D as well as decreased glycosylation of NKG2D and CD70. Decreased NKG2D expression on CD8 T cells and NK cells is the most consistent diagnostic finding of XMEN (Ravell et al., 2020a). Unfortunately, our patients were not available for NKG2D protein evaluation. *In vitro* studies also proved the impaired EBV-specific cytotoxic function on both CD8 T-cells and NK cells of XMEN patients (Chaigne-Delalande et al., 2013; Li et al., 2014; Ravell et al., 2020b). The earliest patients were identified based on persistently high EBV levels and inverted CD4/CD8 ratios (Chaigne-Delalande et al., 2013; Li et al., 2014). Additionally, young children without persistent EBV viremia with an immune phenotype similar to that of EBV-infected patients have been identified (Ravell et al., 2020b). In a review article involving 36 XMEN patients, 1/3 of them developed EBV-driven lymphoproliferation disease or lymphoma, with the age at diagnosis ranging from 5 to 57 years. And five were reported to experience more than one malignancy in their life (Ravell et al., 2020a). A young adult subsequently developed an EBV-negative liposarcoma after the successful treatment of Hodgkin’s lymphoma (Dimitrova et al., 2019). P1 in this study developed non-Hodgkin’s lymphoma at 4 years, and he is now of complete remission after chemotherapy.

Patients with XMEN had a higher predisposition to infectious diseases. Besides persistent EBV viremia, other infections such as CMV, HSV, herpes zoster, and streptococcal infections have also been recorded in XMEN (Li et al., 2014; Patiroglu et al., 2015; Hoyos-Bachiloglu et al., 2020). In this study, all patients were negative for CMV, rubella virus, parvovirus B19, and HIV. To comprehensively evaluate the potential infectious pathogens, we used a probe-based qPCR method to identify the presence of pathogenic microbes in stored whole blood samples. No pathogens other than EBV were detected, although P1 had a history of transient HSV infection. P6 was referred to clinicians for recurrent upper respiratory tract infection, oral ulcers and neutropenia. He was negative for EBV serology and EBV-DNA, and the pathogen detection result was also negative. However, two EBV-negative patients were reported to develop EBV infection during follow-up (Ravell et al., 2020b); therefore, routine checks for EBV infection would be needed for P6 in later life. We also applied metagenomic sequencing for sequence-based identification of pathogenic microbes, including viruses, bacteria, fungi, and parasites, in liver biopsy tissues from P4 and P5 and obtained negative results.

We evaluated the immune phenotypes in our patients and observed low IgA and IgG levels, inverted CD4/CD8 ratios and increased numbers of B cells. P1 underwent lymphocyte subpopulation examination, and elevated αβDNTs was observed, which was reported in 95% of patients with XMEN (Ravell et al., 2020b). All patients in our study had characteristic peripheral blood abnormalities, and they also presented with typical immunologic symptoms. Two patients with *MAGT1* variants were reported to present with different phenotypes of intellectual and developmental disability. They were subsequently diagnosed as congenital glycosylation defects (Blommaert et al., 2019). The finding of selective NLG defect in XMEN disease expanded the clinical presentations to multisystem abnormalities, including hepatic and neurological abnormalities (Ravell et al., 2020b). Taken together, these findings highlight the clinical diversity observed in XMEN patients.

Since its first report in 2011, XMEN disease has been reported in a total of 45 patients aged between 18 months and 58 years. All reported patients were detected P/LP variants in the *MAGT1* gene (Li et al., 2011; Chaigne-Delalande et al., 2013; Li et al., 2014; Dhalla et al., 2015; Patiroglu et al., 2015; Brigida et al., 2017; He et al., 2018; Blommaert et al., 2019; Dimitrova et al., 2019; Hoyos-Bachiloglu et al., 2020; Klinken et al., 2020; Ravell et al., 2020b; Brault et al., 2021). In total, 30 P/LP variants have been reported, including 3 novel variants identified in this study. These variants are distributed over all exons of the gene, except for exon 10 (Figure 3). There are 12 stop-gain, 10 frameshift, 2 splicing, 2 missense variants, and 4 gross deletions in *MAGT1*; this finding indicates a loss-of-function mechanism of this gene. Of note, the missense variant c.1068A > C was detected in a patient characterized by developmental disability (Blommaert et al., 2019). Another missense variant c.97A > T was observed in a patient suffered from EBV-driven cutaneous lymphoproliferation, three times of distinct lymphomas, and multiple infection histories (Klinken et al., 2020). The most common variant, c.409C > T, appeared in five patients from four unrelated families (Ravell et al., 2020b). Another common variant, c.991C > T, was detected in P4 and P5 from family 4, in two reported patients with classical XMEN (Ravell et al., 2020b; Brault et al., 2021), and in another case with a variable phenotype of developmental disability (Blommaert et al., 2019). Therefore, XMEN appears to exhibit variable severity of clinical phenotypes even with identical genotypes. There do not appear to be specific genotype-phenotype correlations among individuals with the disease.

Currently, there lack of effective treatments for XMEN. IVIG combined with antibiotic therapy may reduce opportunistic infections. *In vitro* studies have revealed that MAGT1 deficiency abrogates magnesium influx into T cells. However, oral magnesium supplementation has not proven successful improvement in patients (Chaigne-Delalande et al., 2013; Li et al., 2014; Klinken et al., 2020; Chauvin et al., 2021). Allogeneic HSCT has also been applied to treat XMEN; the disease phenotype reversed in two out of five patients, and three died of transplant-related complications (Li et al., 2014; Dimitrova et al., 2019). Of note, all patients who received HSCT presented with thrombocytopenia and significant hemorrhage after transplantation; this result may suggest a qualitative platelet defect in XMEN patients. Considering the unpredictable complications of HSCT and the stable clinical conditions of our six patients, none of them underwent HSCT. Recently, a pre-clinical study demonstrated that *ex vivo* correcting autologous T and NK cells from XMEN patients through *MAGT1* mRNA electroporation can restore NKG2D receptor expression and rescue cytotoxic activity to normal levels (Brault et al., 2021). This highly efficient method avoids genomic integration and the risks of alloimmunity, supporting cell therapy as an attractive immunologic cure for this monogenetic disease.

In this study, we diagnosed six pediatric XMEN cases predominantly based on genetic testing results. Five patients were referred to the center for pediatric liver diseases, and immunological phenotypes were not evaluated until the molecular diagnosis was made. The combined data from clinical phenotypes and genetic testing suggested a diagnosis of XMEN disease. XMEN is a rare, underdiagnosed disease with manifestations ranging from immunodeficiency and immune dysregulation, to extra-immune symptoms. Some patients were not diagnosed until adulthood because of the insidious onset and indolent course of the disease (Dhalla et al., 2015; Ravell et al., 2020a; Ravell et al., 2020b). To date, 48.9% (22/45) of the patients were diagnosed in their childhood (<18 years of age). The clinical and immunological features of 18 patients with detailed records (Li et al., 2011; Chaigne-Delalande et al., 2013; Li et al., 2014; Dhalla et al., 2015; Patiroglu et al., 2015; Brigida et al., 2017; He et al., 2018; Blommaert et al., 2019; Dimitrova et al., 2019; Hoyos-Bachiloglu et al., 2020; Klinken et al., 2020; Ravell et al., 2020b; Brault et al., 2021) are summarized in Supplementary Table S2. Long-term follow-up is necessary in pediatric patients as the clinical and immunological phenotypes of XMEN may evolve with age. Most cases developed EBV-associated malignancies in their second and third decades of life (Ravell et al., 2020a). P1 was the first diagnosed case and was followed up for 2 years. The patient achieved complete remission of lymphoma for 7 years. Patients may have brain atrophy that is more significant than expected for their age or white matter abnormalities consistent with leukoencephalopathy (Ravell et al., 2020b). Cavum septum pellucidum was found in half patients with XMEN, with a significantly higher rate than that in the general population (Ravell et al., 2020b). Further management and treatment should be modified according to the symptoms of each patient.

In conclusion, this study expands our understanding of the clinical and immunological features as well as the mutational spectrum of XMEN disease. The combined immunodeficiency should be suspected in male patients with abnormal LFTs, chronic EBV infection and EBV-associated lymphoproliferative disease. Genetic diagnosis with *MAGT1* will be crucial for the management and follow-up of the patients, even for effective clinical trials of potential therapies.

## Data Availability

The datasets for this article are not publicly available due to concerns regarding participant/patient anonymity. Requests to access the datasets should be directed to the corresponding authors.
