# Supplementary material for: Further Delineation of the Spectrum of XMEN Disease in Six Chinese Pediatric Patients
Source: Front Genet. 2022 Jan 25;13:768000. doi: 10.3389/fgene.2022.768000 (PMC8821886; doi:10.3389/fgene.2022.768000)
Supplement: Supplementary file 1 [file Table1.pdf]

Supplementary Table 1. Lymphocyte subpopulations of patient 1 with *MAGT1 variant*s

Items	Patient 1
Age at testing	12y11m
CD19 (%)	6.78↓(8.84-17.76)
Naive B (%)	72.7 (53.78-78.64)
Memory B (%)	6.07↓(7.15-23.10)
Transitional B (%)	6.37 (1.38-9.42)
Plasmablasts (%)	0.11↓(0.49-7.06)
CD3 (%)	59.07 (56.84-75.02)
CD4 (%)	18.82↓(22.25-39.00)
CD4 Naive (%)	64.74 (39.50-66.26)
CD4 CM (%)	28.90 (25.34-49.90)
CD4 EM (%)	5.53 (4.68-15.70)
CD8 (%)	30.41 (21.91-36.80)
CD8 Naive (%)	83.94↑(35.34-72.32)
CD8 CM (%)	11.28 (10.96-31.00)
CD8 EM (%)	1.36↓(2.38-15.84)
CD8 TEMRA (%)	3.41↓（5.08-31.24）
DNT (%)	16.77
αβDNT (%)	3.95↑ (0.61-2.31)
γδ T (%)	6.88 (6.55-20.28)

The number in the round bracket presents the age-specific reference percentage according to reference values for peripheral blood lymphocyte subsets of healthy children in China

CM: Central memory; EM, Effector memory; TEMRA: Terminal effector memory cytotoxic T cells; DNT, CD4 and CD8 double-negative T cell; αβDNT, TCRαβ+ CD4 and CD8 double-negative T cell.
