# Supplementary material for: Further Delineation of the Spectrum of XMEN Disease in Six Chinese Pediatric Patients
Source: Front Genet. 2022 Jan 25;13:768000. doi: 10.3389/fgene.2022.768000 (PMC8821886; doi:10.3389/fgene.2022.768000)
Supplement: Supplementary file 2 [file Table2.docx]

**Supplementary Table 2. Clinical and genetic features of eighteen reported pediatric XMEN cases in the literature**

| patient ID | #1-1 | #1-2 | #2-1 | #3-1 | #4-1 | #5-1 | #6-1 | #7-1 | #8-1 | #9-1 | #10-1 | #11-1 | #12-1 | #13-1 | #14-1 | #14-2 | #15-1 | #16-1 |
| --- | --- | --- | --- | --- | --- | --- | --- | --- | --- | --- | --- | --- | --- | --- | --- | --- | --- | --- |
| age at diagnosis | 7yrs | 3yrs | 4yrs | 16yrs | 16yrs | 13yrs | 15yrs | 16yrs | 2yrs | 17yrs | 15yrs | 13yrs | 11yrs | 17yrs | 32m | 18m | 10yrs | 6yrs |
| cDNA variant (NM_032121.5) | c.859_997del139 | c.859_997del139 | c.236G>A | c.409C>T | c.598delC | c.712C>T | Partial gene deletion exons 3-10 | c.900_901dupAA | c.223C>T | c.555dupA | c.737_738insGA | c.1068A>C | c.991C>T | c.938T>G | c.472delG | c.472delG | c.771T>A | Partial gene deletion exons 2–10 |
| protein variant | p.(Asn287*) | p.(Asn287*) | p.(Trp79*) | p.(Arg137*) | p.(Arg200Glyfs*13) | p.(Arg238*) | / | p.(Thr301Lysfs*14) | p.(Gln75*) | p.(Tyr186Ilefs*2) | p.(Phe246Leufs*18) | p.(Lys356Asn) | p.(Arg331*) | p.(Leu313*) | p.(Asp158Metfs*6) | p.(Asp158Metfs*6) | p.(Cys257*) | / |
| elevated liver enzymes | + | + | NA | + | + | + | + | + | + | + | NA | NA | NA | NA | - | NA | NA | NA |
| recurrent ear and sinopulmonary infections | + | + | NA | - | - | + | - | + | + | + | NA | NA | NA | NA | + | + | NA | + |
| EBV viremia | + | + | + | + | + | + | + | + | - | + | + | - | - | + | - | - | - | + |
| lymphoproliferation or neoplasia | - | - | - | B-cell LPD | Burkitt’s lymphoma | B-cell LPD | Hodgkin lymphoma | EBV-positive LPD | B-cell LPD | Hodgkin lymphoma | Hodgkin’s lymphoma | - | - | - | - | - | Castleman disease | Kaposis sarcoma |
| developmental disability | - | - | - | - | - | - | - | - | - | - | - | + | + | - | - | - | - | + |
| decreased NKG2D expression on NK and CD8 T cells | + | + | + | + | + | + | + | + | + | + | + | NA | NA | + | + | + | + | + |
| elevated B cells | + | + | NA | + | + | + | + | + | + | + | - | NA | NA | NA | + | + | + | + |
| inverted CD4:CD8 ratio | + | + | + | + | + | + | + | + | - | + | + | NA | NA | - | + | + | + | NA |
| CD4 lymphopenia | + | + | - | - | + | - | - | + | + | + | - | NA | NA | - | + | + | + | + |
| low IgA | + | + | - | + | + | + | + | + | + | - | NA | NA | NA | NA | + | NA | NA | + |
| low IgG | + | + | - | + | + | + | + | + | + | - | NA | NA | NA | NA | - | NA | NA | + |
| thrombocytopenia | + | + | NA | + | + | + | + | + | + | + | + | NA | NA | NA | - | - | - | NA |
| transient neutropenia | + | + | NA | + | + | + | + | + | + | - | NA | NA | NA | NA | - | - | - | NA |
| HSV infection | + | + | - | - | + | - | - | - | - | - | - | - | - | - | - | - | - | - |
| CMV infection | - | - | - | - | - | - | - | - | + | + | + | - | - | - | - | - | - | - |
| outcome | alive | alive | NA | died (HSCT) | alive (mRNA) | alive (HSCT) | alive | alive (HSCT) | NA | died (HSCT) | alive | alive | alive | alive | alive | alive | alive (HSCT) | alive |
| reference | (Li et al., 2011; Chaigne-Delalande et al., 2013; Ravell et al., 2020) | (Li et al., 2011; Chaigne-Delalande et al., 2013; Ravell et al., 2020) | (Chaigne-Delalande et al., 2013; Li et al., 2014; Ravell et al., 2020) | (Chaigne-Delalande et al., 2013; Li et al., 2014; Ravell et al., 2020) | (Chaigne-Delalande et al., 2013; Li et al., 2014; Ravell et al., 2020; Brault et al., 2021) | (Dhalla et al., 2015; Ravell et al., 2020) | (Ravell et al., 2020) | (Dimitrova et al., 2019; Ravell et al., 2020) | (Hoyos-Bachiloglu et al., 2020; Ravell et al., 2020) | (Patiroglu et al., 2015; Ravell et al., 2020) | (Hoyos-Bachiloglu et al., 2020) | (Blommaert et al., 2019) | (Blommaert et al., 2019) | (Blommaert et al., 2019) | (He et al., 2018) | (He et al., 2018) | (Klinken et al., 2020) | (Brigida et al., 2017) |

*LPD*: lymphoproliferative disease; *HSCT*: hematopoietic stem cell transplant; *NA*: not available

**Reference**

Blommaert, E., Peanne, R., Cherepanova, N.A., Rymen, D., Staels, F., Jaeken, J., et al. (2019). Mutations in MAGT1 lead to a glycosylation disorder with a variable phenotype. *Proc Natl Acad Sci U S A* 116(20)**,** 9865-9870. doi: 10.1073/pnas.1817815116.

Brault, J., Meis, R.J., Li, L., Bello, E., Liu, T., Sweeney, C.L., et al. (2021). MAGT1 messenger RNA-corrected autologous T and natural killer cells for potential cell therapy in X-linked immunodeficiency with magnesium defect, Epstein-Barr virus infection and neoplasia disease. *Cytotherapy* 23(3)**,** 203-210. doi: 10.1016/j.jcyt.2020.08.013.

Brigida, I., Chiriaco, M., Di Cesare, S., Cittaro, D., Di Matteo, G., Giannelli, S., et al. (2017). Large Deletion of MAGT1 Gene in a Patient with Classic Kaposi Sarcoma, CD4 Lymphopenia, and EBV Infection. *J Clin Immunol* 37(1)**,** 32-35. doi: 10.1007/s10875-016-0341-y.

Chaigne-Delalande, B., Li, F.Y., O'Connor, G.M., Lukacs, M.J., Jiang, P., Zheng, L., et al. (2013). Mg2+ regulates cytotoxic functions of NK and CD8 T cells in chronic EBV infection through NKG2D. *Science* 341(6142)**,** 186-191. doi: 10.1126/science.1240094.

Dhalla, F., Murray, S., Sadler, R., Chaigne-Delalande, B., Sadaoka, T., Soilleux, E., et al. (2015). Identification of a novel mutation in MAGT1 and progressive multifocal leucoencephalopathy in a 58-year-old man with XMEN disease. *J Clin Immunol* 35(2)**,** 112-118. doi: 10.1007/s10875-014-0116-2.

Dimitrova, D., Rose, J.J., Uzel, G., Cohen, J.I., Rao, K.V., Bleesing, J.H., et al. (2019). Successful Bone Marrow Transplantation for XMEN: Hemorrhagic Risk Uncovered. *J Clin Immunol* 39(1)**,** 1-3. doi: 10.1007/s10875-018-0573-0.

He, T.Y., Xia, Y., Li, C.G., Li, C.R., Qi, Z.X., and Yang, J. (2018). [X-linked immunodeficiency with magnesium defect, Epstein-Barr virus infection, and neoplasia: report of a family and literature review]. *Zhonghua Er Ke Za Zhi* 56(1)**,** 48-52. doi: 10.3760/cma.j.issn.0578-1310.2018.01.013.

Hoyos-Bachiloglu, R., Concha, S., Sepulveda, P., Campos, R., Perez-Mateluna, G., King, A., et al. (2020). The Many Faces of XMEN Disease, Report of Two Patients with Novel Mutations. *J Clin Immunol* 40(2)**,** 415-417. doi: 10.1007/s10875-020-00746-1.

Klinken, E.M., Gray, P.E., Pillay, B., Worley, L., Edwards, E.S.J., Payne, K., et al. (2020). Diversity of XMEN Disease: Description of 2 Novel Variants and Analysis of the Lymphocyte Phenotype. *J Clin Immunol* 40(2)**,** 299-309. doi: 10.1007/s10875-019-00732-2.

Li, F.Y., Chaigne-Delalande, B., Kanellopoulou, C., Davis, J.C., Matthews, H.F., Douek, D.C., et al. (2011). Second messenger role for Mg2+ revealed by human T-cell immunodeficiency. *Nature* 475(7357)**,** 471-476. doi: 10.1038/nature10246.

Li, F.Y., Chaigne-Delalande, B., Su, H., Uzel, G., Matthews, H., and Lenardo, M.J. (2014). XMEN disease: a new primary immunodeficiency affecting Mg2+ regulation of immunity against Epstein-Barr virus. *Blood* 123(14)**,** 2148-2152. doi: 10.1182/blood-2013-11-538686.

Patiroglu, T., Haluk Akar, H., Gilmour, K., Unal, E., Akif Ozdemir, M., Bibi, S., et al. (2015). A case of XMEN syndrome presented with severe auto-immune disorders mimicking autoimmune lymphoproliferative disease. *Clin Immunol* 159(1)**,** 58-62. doi: 10.1016/j.clim.2015.04.015.

Ravell, J.C., Matsuda-Lennikov, M., Chauvin, S.D., Zou, J., Biancalana, M., Deeb, S.J., et al. (2020). Defective glycosylation and multisystem abnormalities characterize the primary immunodeficiency XMEN disease. *J Clin Invest* 130(1)**,** 507-522. doi: 10.1172/JCI131116.
